# Supplementary material for: Regional trends, spatial patterns and determinants of health facility delivery among women of reproductive age in Nigeria: A national population based cross-sectional study
Source: PLoS One. 2024 Oct 16;19(10):e0312005. doi: 10.1371/journal.pone.0312005 (PMC11482673; doi:10.1371/journal.pone.0312005)
Supplement: S1 File — (PDF) [file pone.0312005.s001.pdf]

```
clear all
set more off
set maxvar 15000
set memory 15000
use "C:\Users\USER\Desktop\Articles\Tope Olubodun\Manuscript2\NGIR7AFL.dta"

********WEIGHTING******************
gen wgt = v005/1000000

svyset[pw=wgt],psu(v001) strata(v022) singleunit(center)


********SAMPLE SIZE******************
drop if v201 == 0


************ RECODE MARITAL STATUS****************
tab v502,m

recode v502 (0 2 = 1 "unmarried")(1 = 2 "married"), gen (mstatus)

tab mstatus,m


*********** HF DELIVERY ***********************
tab m15_1,m

drop if m15_1 == .

drop if m15_1 == 99

tab m15_1,m

recode m15_1 (11 12 96 = 0 " no hf delivery")(21 22 23 26 31 36 = 1 " hf delivery"), gen (hfdelivery)

tab hfdelivery,m


*************************************************************************
*************************************************************************
************ INDEPENDENT VARIABLES****************************************

*******RECODE AGE AT LAST CHILDBIRTH************

gen agebirth =(b3_01 - v011)/12

tab agebirth,m

recode agebirth (11/19.9999 = 1 "15-19") (20/29.9999 = 2 "20-29") (30/39.9999 = 3 "30-39")
(40/49.9999 = 4 "40-49"), gen (cat_agebirth)
```

```
tab cat_agebirth,m


**********RECODE RELIGION**************
tab v130,m

recode v130 (1/2 = 1 "christianity")(3 = 2 "Islam")(4 96 99 . = 3 "traditional/others"), gen (religion)

tab religion,m

*************RECODE ETHNICITY ***********************

gen ethnless98 = v131 if v131 < 98

recode ethnless98 (1 4 5 7 9 96 = 4 "others")(2 3 8 = 1 "haua/fulani/kanuri")(10 = 2 "yoruba")(6 = 3 "igbo"), gen (ethnicity)


*************EDUCATION*****************

rename v106 educ

tab educ, m

*********EMPLOYMENT*****************

tab v714,m

recode v714 (0 9 . = 0 "unemployed") (1 = 1 "employed"), gen (employm)

tab employ,m

*********** WEALTH INDEX*************

xtile wealthindex = v191, nq(3)

la def wealthindex 1 poor 2 middle 3 rich

la val wealthindex wealthindex

tab wealthindex, m


***************RECODE MASS MEDIA EXPOSURE*****************

**** v157 newspaper*****

tab v157,m

tab v157,m
```

```
recode v157 (0/1 = 0 "poor read") (2/3 = 1 "good read"), gen (newspaper)

tab newspaper,m

******* v158 radio************

tab v158,m

recode v158 (0/1 = 0 "poor listen") (2/3 = 1 "good listen"), gen (radio)

tab radio,m


******* v159 television**********

tab v159,m

recode v159 (0/1 = 0 "poor watch") (2/3 = 1 "good watches"), gen (television)

tab television,m

***** massmedia******************************************

gen massmedia = newspaper + radio + television

tab massmedia,m

recode massmedia (0 = 0 "no/poor mass media") (1/3 = 1 "frequent mass media"), gen
(cat_massmedia)

tab cat_massmedia, m

*********RECODE WANTED PREGNANCY*******************

tab m10_1,m

recode m10_1 (1 = 1 "yes")(2 3 9 . = 2 "no"), gen (wantedpreg)

tab wantedpreg,m

**********RECODE NUMBER OF CHILDBIRTHS****************

tab v201,m

recode v201 (1/2 = 1 "1-2")(3/4 = 2 "3-4")(5/20 = 3 "5-17"), gen (no_child)

tab no_child,m

*********DISTANCE TO HF*********

tab v467d,m
```

```
rename v467d distancetohf

tab distancetohf,m

*********COMPANION*********

tab v467f, m

rename v467f companion

tab companion,m

***********RECODE DECIDES ON HEALTHCARE**********************

tab v743a,m

recode v743a (1 2 = 1 "participates")(4 5 6 . = 2 "does not participate"), gen (decideshc)

tab decideshc,m

**********RECODE HUSBANDS EDUCATION  **********************

gen husbeduc = v701 if v701 < 8

tab husbeduc,m

******** PLACE OF RESIDENCE*************

rename v025 place_res

tab place_res, m

******** REGION*************

rename v024 region

*************ANC VISITS**************
tab m14_1,m

gen anc = m14_1 if m14_1 < 98

tab anc,m

recode anc (0/3 = 0 "less than 4")(4/20 = 1 "at least 4"), gen (no_anc)

tab no_anc, m

***************COMMUNITY POVERTY***************

gen commpov = 0
```

```
replace commpov = 1 if v190 == 1

bysort v001:egen commpov2 = mean (commpov)

xtile commpov3 = commpov2, nq(3)


*************COMMUNITY EDUCATION******************

recode educ (0/1 = 1 "no educ/primary")(2/3 = 2 "secon/higher"), gen (educ2grps)

gen commeduc = 0

replace commeduc = 1 if educ2grps ==2

bysort v001:egen commeduc2 = mean (commeduc)

xtile commeduc3 = commeduc2, nq(3)


**********COMMUNITY DISTANCE TO HF*************

gen commdist = 0

replace commdist = 1 if distancetohf == 1

bysort v001:egen commdist2 = mean (commdist)

xtile commdist3 = commdist2, nq(3)


****************************************************************************
******************************************************************************
******************************************************************************
********************FREQUENCY TABLES****************************************

svy: tab hfdelivery, per obs

svy: tab cat_agebirth, per obs

svy: tab mstatus, per obs

svy: tab religion, per obs

svy: tab ethnicity, per obs

svy: tab educ, per obs

svy: tab employ,per obs
```

svy: tab wealthindex, per obs

svy: tab cat_massmedia, per obs

svy: tab wantedpreg, per obs

svy: tab no_child, per obs

svy: tab no_anc, per obs

svy: tab companion, per obs

svy: tab decideshc, per obs

svy: tab husbeduc, per obs

svy: tab place_res, per obs

svy: tab region, per obs

svy: tab commpov3, per obs

svy: tab commeduc3, per obs

svy: tab commdist3, per obs

*****##******BIVARIATE ANALYSIS ***
****
****
****

svy: tab cat_agebirth hfdelivery, percent row obs
svy: tab mstatus hfdelivery, percent row obs
svy: tab religion hfdelivery, percent row obs
svy: tab ethnicity hfdelivery, percent row obs
svy: tab educ hfdelivery, percent row obs
svy: tab employ hfdelivery, percent row obs
svy: tab wealthindex hfdelivery, percent row obs
svy: tab cat_massmedia hfdelivery, percent row obs
svy: tab wantedpreg hfdelivery, percent row obs
svy: tab no_child hfdelivery, percent row obs
svy: tab no_anc hfdelivery, percent row obs
svy: tab companion hfdelivery, percent row obs
svy: tab husbeduc hfdelivery, percent row obs
svy: tab decideshc hfdelivery, percent row obs
svy: tab place_res hfdelivery, percent row obs
svy: tab region hfdelivery, percent row obs

svy: tab commpov3 hfdelivery, percent row obs
svy: tab commeduc3 hfdelivery, percent row obs
svy: tab commdist3 hfdelivery, percent row obs

***Crude odd ratios
svy: logistic hfdelivery i.cat_agebirth
svy: logistic hfdelivery i.mstatus
svy: logistic hfdelivery i.religion
svy: logistic hfdelivery i.ethnicity
svy: logistic hfdelivery i.educ
svy: logistic hfdelivery i.employ
svy: logistic hfdelivery i.wealthindex
svy: logistic hfdelivery i.cat_massmedia
svy: logistic hfdelivery i.wantedpreg
svy: logistic hfdelivery i.no_child
svy: logistic hfdelivery i.no_anc
svy: logistic hfdelivery i.companion
svy: logistic hfdelivery i.husbeduc
svy: logistic hfdelivery i.decideshc
svy: logistic hfdelivery i.place_res
svy: logistic hfdelivery i.region
svy: logistic hfdelivery i.commpov3
svy: logistic hfdelivery i.commeduc3
svy: logistic hfdelivery i.commdist3

************* VIF **********************************
logistic hfdelivery i.cat_agebirth i.mstatus i.religion i.ethnicity i.educ i.employ i.wealthindex i.cat_massmedia ib2.wantedpreg i.no_child i.no_anc ib2.companion ib2.decideshc i.husbeduc i.place_res i.region i.commpov3 i.commeduc3 i.commdist3

vif, uncentered

********* EMPTY MODEL REGRESSION*******************
xtmelogit hfdelivery || v001: || v002:, or variance

estat ic

estat icc

****** INDIVIDUAL LOGISTIC REGRESSION
****
xtmelogit hfdelivery i.cat_agebirth i.religion i.ethnicity i.educ i.employ i.wealthindex i.cat_massmedia ib2.wantedpreg i.no_child i.no_anc ib2.companion ib2.decideshc i.husbeduc|| v001: || v002:, or variance

estat ic

estat icc

****** COMMUNITY LOGISTIC REGRESSION
****
xtmelogit hfdelivery i.place_res i.region i.commpov3 i.commeduc3 i.commdist3 || v001: || v002:, or variance

estat ic

estat icc
