# Supplementary material for: Regional trends, spatial patterns and determinants of health facility delivery among women of reproductive age in Nigeria: A national population based cross-sectional study
Source: PLoS One. 2024 Oct 16;19(10):e0312005. doi: 10.1371/journal.pone.0312005 (PMC11482673; doi:10.1371/journal.pone.0312005)

PLOS ONE

Regional Trends, Spatial Patterns and Determinants of Health Facility Delivery Among Women of Reproductive Age in Nigeria: A National Population Based Cross-Sectional Study

--Manuscript Draft--

**Manuscript Number:**	PONE-D-23-37668
**Article Type:**	Research Article
**Full Title:**	Regional Trends, Spatial Patterns and Determinants of Health Facility Delivery Among Women of Reproductive Age in Nigeria: A National Population Based Cross-Sectional Study
**Short Title:**	Regional Trends, Spatial Patterns and Determinants of Health Facility Delivery Among Women in Nigeria
**Corresponding Author:**	Tope Olubodun, M.D, M.P.H.<br>Federal Medical Centre Abeokuta<br>Abeokuta, Ogun NIGERIA
**Keywords:**	Health facility delivery; Institutional delivery; Facility based delivery; determinants; trends; Spatial patterns; Nigeria
**Abstract:**	Background: Globally, about 810 women die daily from pregnancy and childbirth complications, and the burden is highest in Africa. The United Nations sustainable development goal has a maternal mortality ratio (MMR) target of 70 per 100,000 live births by 2030. Nigeria, the largest country in Africa, has an MMR of 512 per 100,000 live births, thus there is need for intensified efforts to reduce maternal deaths in the country. Proper utilisation of maternal health services including health facilities for delivery is crucial to achieving this. This study assesses the regional trends, spatial patterns and determinants of health facility delivery among women of reproductive age in Nigeria.<br>Methods: This is a weighted secondary analysis of the Nigerian Demographic and Health Survey (NDHS). The sample included women who had a live birth in the preceding 5 years of the NDHS 2008, 2013 and 2018. Bivariate analysis and multilevel logistic regression were carried out to assess the determinants of health facility delivery. Trends analysis was done using bar graphs and spatial analysis showed the distribution of health facility delivery by State.<br>Results: Forty-one percent of women delivered their last child in a health facility. The proportion of women who delivered at a health facility increased from 2008 to 2018 for all regions, with exception of the South-south region. Determinants of facility-based delivery include; ethnicity, level of education, wealth index, exposure to mass media (AOR 1.34; 95% CI 1.20 – 1.50), number of childbirths, number of antenatal visits (AOR 4.03; 95% CI 3.51 – 4.62), getting a companion to go the health facility (AOR 0.84; 95% CI 0.72 – 0.98), community level poverty, community level of female education, community distance to health facility, and geographical region.<br>Conclusion: There is an urgent need to deploy appropriate strategies and programme to improve health facility delivery in Nigeria.
**Order of Authors:**	Tope Olubodun, MD, MPH.
	Olorunfemi Akinbode Ogundele
	Turnwait Otu Michael
	Oluyemi A Okunlola
	Ayodeji Bamidele Olubodun
	Semiu Adebayo Rahman
**Additional Information:**	
**Question**	**Response**
**Financial Disclosure**	The author(s) received no specific funding for this work.

Powered by Editorial Manager® and ProduXion Manager® from Aries Systems Corporation

Enter a financial disclosure statement that describes the sources of funding for the work included in this submission. Review the submission guidelines for detailed requirements. View published research articles from *PLOS ONE* for specific examples.<br><br>This statement is required for submission and **will appear in the published article** if the submission is accepted. Please make sure it is accurate.<br><br>**Funded studies**<br>Enter a statement with the following details:<br>• Initials of the authors who received each award<br>• Grant numbers awarded to each author<br>• The full name of each funder<br>• URL of each funder website<br>• Did the sponsors or funders play any role in the study design, data collection and analysis, decision to publish, or preparation of the manuscript?<br><br>Did you receive funding for this work?	
**Competing Interests**<br><br>Use the instructions below to enter a competing interest statement for this submission. On behalf of all authors, disclose any competing interests that could be perceived to bias this work—acknowledging all financial support and any other relevant financial or non-financial competing interests.<br><br>This statement is required for submission and **will appear in the published article** if the submission is accepted. Please make sure it is accurate and that any funding sources listed in your Funding Information later in the submission form are also declared in your Financial Disclosure statement.	The authors have declared that no competing interests exist

View published research articles from [*PLOS ONE*](#) for specific examples.<br><br>**NO authors have competing interests**<br><br>Enter: *The authors have declared that no competing interests exist.*<br><br>**Authors with competing interests**<br><br>Enter competing interest details beginning with this statement:<br><br>*I have read the journal's policy and the authors of this manuscript have the following competing interests: [insert competing interests here]*<br><br>* typeset	
**Ethics Statement**<br><br>Enter an ethics statement for this submission. This statement is required if the study involved:<br><br>• Human participants<br>• Human specimens or tissue<br>• Vertebrate animals or cephalopods<br>• Vertebrate embryos or tissues<br>• Field research<br><br>Write "N/A" if the submission does not require an ethics statement.<br><br>General guidance is provided below. Consult the submission guidelines for detailed instructions. **Make sure that all information entered here is included in the Methods section of the manuscript.**	Being a secondary data analysis, ethical approval was not required for this study. We registered and obtained permission to download the datasets from the measure DHS website. However, in the primary studies – The NDHS 2008, 2013, and 2018, the survey protocols were approved after review by the ICF Institutional Review Board and the National Health Research Ethics Committee of Nigeria (NHREC). Informed consent was obtained, and all methods were performed in accordance with the Declaration of Helsinki.

Powered by Editorial Manager® and ProduXion Manager® from Aries Systems Corporation

**Format for specific study types**<br><br>**Human Subject Research (involving human participants and/or tissue)**<br>• Give the name of the institutional review board or ethics committee that approved the study<br>• Include the approval number and/or a statement indicating approval of this research<br>• Indicate the form of consent obtained (written/oral) or the reason that consent was not obtained (e.g. the data were analyzed anonymously)<br><br>**Animal Research (involving vertebrate animals, embryos or tissues)**<br>• Provide the name of the Institutional Animal Care and Use Committee (IACUC) or other relevant ethics board that reviewed the study protocol, and indicate whether they approved this research or granted a formal waiver of ethical approval<br>• Include an approval number if one was obtained<br>• If the study involved *non-human primates*, add *additional details* about animal welfare and steps taken to ameliorate suffering<br>• If anesthesia, euthanasia, or any kind of animal sacrifice is part of the study, include briefly which substances and/or methods were applied<br><br>**Field Research**<br><br>Include the following details if this study involves the collection of plant, animal, or other materials from a natural setting:<br>• Field permit number<br>• Name of the institution or relevant body that granted permission	
**Data Availability**<br><br>Authors are required to make all data underlying the findings described fully available, without restriction, and from the time of publication. PLOS allows rare exceptions to address legal and ethical concerns. See the PLOS Data Policy and FAQ for detailed information.	No - some restrictions will apply

Powered by Editorial Manager® and ProduXion Manager® from Aries Systems Corporation

A Data Availability Statement describing where the data can be found is required at submission. Your answers to this question constitute the Data Availability Statement and **will be published in the article**, if accepted.<br><br>**Important:** Stating 'data available on request from the author' is not sufficient. If your data are only available upon request, select 'No' for the first question and explain your exceptional situation in the text box.<br><br>Do the authors confirm that all data underlying the findings described in their manuscript are fully available without restriction?	
**Describe where the data may be found in full sentences. If you are copying our sample text, replace any instances of XXX with the appropriate details.**<br><br>• If the data are **held or will be held in a public repository**, include URLs, accession numbers or DOIs. If this information will only be available after acceptance, indicate this by ticking the box below. For example: *All XXX files are available from the XXX database (accession number(s) XXX, XXX.)*.<br>• If the data are all contained **within the manuscript and/or Supporting Information files**, enter the following: *All relevant data are within the manuscript and its Supporting Information files.*<br>• If neither of these applies but you are able to provide **details of access elsewhere**, with or without limitations, please do so. For example:<br><br>*Data cannot be shared publicly because of [XXX]. Data are available from the XXX Institutional Data Access / Ethics Committee (contact via XXX) for researchers who meet the criteria for access to confidential data.*<br><br>*The data underlying the results presented in the study are available from (include the name of the third party*	The data underlying the results presented in the study are available after registration and request from measure DHS website (https://dhsprogram.com).

Powered by Editorial Manager® and ProduXion Manager® from Aries Systems Corporation

and contact information or URL).<br>• This text is appropriate if the data are owned by a third party and authors do not have permission to share the data.<br><br>* typeset	
Additional data availability information:	

Manuscript Click here to access/download;Manuscript;Manuscript.docx

Title: Regional Trends, Spatial Patterns and Determinants of Health Facility Delivery Among Women of Reproductive Age in Nigeria: A National Population Based Cross-Sectional Study

Short Title: Regional Trends, Spatial Patterns and Determinants of Health Facility Delivery Among Women in Nigeria

Authors

Tope Olubodun[1*], Olorunfemi Akinbode Ogundele[2], Turnwait Otu Michael[3], Oluyemi A. Okunlola[4], Ayodeji Bamidele Olubodun[5], Semiu Adebayo Rahman[6]

1 Department of Community Medicine and Primary Care, Federal Medical Center, Abeokuta, Ogun State, Nigeria
Email: olubodunto pe@gmail.com
Phone number: +2348173353388

2 Department of Community Medicine, University of Medical Sciences, Ondo City, Nigeria

3 Department of Sociology, University of Johannesburg, South Africa

4 Department of Mathematical and Computer Sciences, University of Medical Sciences, Ondo City, Nigeria

5 Olabisi Onabanjo University Teaching Hospital, Nigeria.

6 Department of Demography and Social Statistics, Obafemi Awolowo University, Ile Ife, Osun State, Nigeria.

*Corresponding author (TO)
oluboduntope@gmail.com

Abstract

Background: Globally, about 810 women die daily from pregnancy and childbirth complications, and the burden is highest in Africa. The United Nations sustainable development goal has a maternal mortality ratio (MMR) target of 70 per 100,000 live births by 2030. Nigeria, the largest country in Africa, has an MMR of 512 per 100,000 live births, thus there is need for intensified efforts to reduce maternal deaths in the country. Proper utilisation of maternal health services including health facilities for delivery is crucial to achieving this. This study assesses the regional trends, spatial patterns and determinants of health facility delivery among women of reproductive age in Nigeria.

Methods: This is a weighted secondary analysis of the Nigerian Demographic and Health Survey (NDHS). The sample included women who had a live birth in the preceding 5 years of the NDHS 2008, 2013 and 2018. Bivariate analysis and multilevel logistic regression were carried out to assess the determinants of health facility delivery. Trends analysis was done using bar graphs and spatial analysis showed the distribution of health facility delivery by State.

Results: Forty-one percent of women delivered their last child in a health facility. The proportion of women who delivered at a health facility increased from 2008 to 2018 for all regions, with exception of the South-south region. Determinants of facility-based delivery include; ethnicity, level of education, wealth index, exposure to mass media (AOR 1.34; 95% CI 1.20 – 1.50), number of childbirths, number of antenatal visits (AOR 4.03; 95% CI 3.51 – 4.62), getting a companion to go the health facility (AOR 0.84; 95% CI 0.72 – 0.98), community level poverty, community level of female education, community distance to health facility, and geographical region.

Conclusion: There is an urgent need to deploy appropriate strategies and programme to improve health facility delivery in Nigeria.

Introduction

Delivery in a health facility by skilled birth attendants and with available life-saving commodities and facilities reduces maternal morbidity and morbidity significantly [1,2]. Health facility delivery also reduces stillbirth rate and neonatal morbidity [3,4]. Recognising the importance of health facility delivery, the World Health Organization (2023) recommends that all births take place at health facilities with the assistance of skilled birth attendants. In 2021, 84% of births worldwide happened in health facilities [5]. The proportion of facility delivery varies greatly among countries. Almost all newborns (99%) are born at a health facility in developed countries [5]. Only 64% of babies in Sub-Saharan Africa (SSA) have skilled birth assistance during delivery [5]. Others give birth at home with the help of inexperienced birth attendants, family members, or self-delivery [6].

In Nigeria, only 41% of Nigerian women deliver in health facilities. [7]. Variations in health facility delivery exist across Nigerian geopolitical zones, ranging from 16% in the Northwest zones to 81% in the Southeast zones [8]. Tackling the challenges associated with health-care delivery is critical, especially in Nigeria, where the crude birth rate is an estimated 38 births per 1000 women [8], with a total fertility rate of 5.3 [8]. Disregarding adequate actions to improve health facility delivery in Nigeria will exacerbate the country's already high maternal mortality rate (MMR) of 512 per 100,000 live births, which makes Nigeria still a long way from meeting the Sustainable Development Goal of fewer than 70 maternal deaths per 100,000 live births by 2030 [9].

To enhance maternal health in Nigeria, the Midwives Service Scheme and the Subsidy Reinvestment and Empowerment Programme (SURE-P) were introduced [10,11]. The Midwives service scheme was introduced in 2009 to improve availability of skilled birth

attendants in rural areas of the country [10]. The program engages retired midwives, unemployed and newly graduated midwives to work temporarily in rural areas. In 2012, SURE-P was introduced in a bid to re-invest fuel subsidy funds into social safety net programs including improving maternal health. SURE – P includes a conditional cash transfer for mothers attending at least four antenatal visits, delivering in a health facility, and also attending postnatal care visits, health facility renovations and staffing, ensuring supply of essential maternal health commodities, and community mobilization through village health workers and community leaders [11]. Both programmes had some successes [10,11], however, in 2018, only 41% of Nigerian women in Nigeria delivered at a health facility [7]

Some studies have been carried out using nationally representative data to assess health facility delivery in Nigeria. Our study adds to the body of knowledge from these studies. Ononokpono et al. identified the determinants of health facility delivery among Nigerian women using data from the 2008 NDHS [12]. Adedokun identified the factors associated with home delivery among Nigerian women and conducted a spatial analysis to capture the locations where home delivery is prevalent in the country using data from the 2013 NDHS [13]. Dahiru et al on the other hand assessed the determinants of antenatal care, health facility delivery and postnatal care among women in Nigeria using the 2018 NDHS, but only included individual level variables in analysis and did not account for the hierarchical nature of the NDHS [14]. Solanke et al. assessed the determinants of health facility delivery with emphasis on community level factors but only examined a few individual factors [15].

Our study is different from these studies as we assess the regional trends, spatial patterns, and determinants of health facility delivery among women of reproductive age in Nigeria. Unlike Ononokpono, we use the most recent DHS to provide more recent estimates. Unlike Adedokun

et al, we examine the regional trends, determinants, and spatial patterns of health facility delivery rather than home delivery. Unlike Dahiru et. al. we use a multilevel approach which takes into account the hierarchical nature of the DHS, and unlike Solanke et al, we include a significant number of individual level factors and community level factors. Our study thus provides recent estimates of the determinants of health facility delivery, examines the trends in health facility delivery over ten years and across the six geopolitical zones, and uses spatial analysis to demonstrate parts of the country with high, medium, and low prevalence of health facility delivery. These findings will provide evidence that can guide policy and programming in maternal health in Nigeria.

Methods

Data Source

Data from Nigeria Demographic and health surveys were used in this study. The trend analysis made use of data from NDHS 2008, NDHS 2013 and NDHS 2018 to analyze the trend of health facility delivery across these years, and across the six geo-political zones. Analysis of the determinants of health facility delivery and the spatial analysis of the distribution of health facility delivery across States was done using data from the most recent NDHS i.e., NDHS 2018. Demographic and Health Surveys (DHS) are nationally representative household surveys that provide data for a wide range of indicators in the areas of population, health, and nutrition [16]. They are usually carried out every five years and the data can be used for monitoring and impact evaluation and research [16].

NDHS uses a two-stage cluster sampling approach to select respondents from rural and urban areas in Nigeria and from the 36 States and the FCT. The primary sampling units (PSU)/clusters are the enumeration areas (EAs) from the 2006 census and the Population and Housing Census of the Federal Republic of Nigeria (NPHC), conducted in 2006 provides the sampling frame [8].

Study variables

Outcome variable

The outcome variable is facility-based delivery of the most recent birth. Births in a public or private health facility was defined as 'utilized health facility delivery' and coded as 1, while those who delivered at home or elsewhere were defined as 'not utilizing health facility delivery and was coded as 0.

Exposure variables

Age at last childbirth was derived by subtracting the date of birth of mother (in century month code CMC) from date of birth of child (in century month code) and dividing by 12. Age group was then categorized as 15 – 19 years, 20 – 29 years, 30 – 39 years, 40 – 49 years. Women that were never in union and those that were formerly in union/living with a man were categorized as 'not married' and those currently in union/living with a man were grouped as 'married'. Religion was categorised as Christianity, Islam, and Traditional religion. Ethnicity was categorised as Hausa/Fulani, Yoruba, Ibo, and Others. Level of education was expressed as no education, primary, secondary, and higher. Respondents' employment status was categorized as 'currently working' and 'not currently working'. Wealth index was generated as a tertile of the wealth index factor score into poor, middle and rich.

Mass media exposure was generated from exposure to television, radio, and newspaper. Mass media exposure was defined as frequent exposure for those with at least once a week exposure to television, radio or newspaper, and No exposure/infrequent exposure for those who had no access to any of these or less than once a week exposure to any of these. Wanted index pregnancy was recoded as 'wanted' or 'not wanted'. Number of childbirths was categorized as 1 – 2, 3 - 4, ≥ 5. Number of antenatal care (ANC) visits was categorized as less than four ANC visits, and at least four ANC visits. Companionship to health facility was categorized as being 'a big problem' and 'not a big problem'. Woman's participation in healthcare decision was recoded as participate and does not participate. Partner's education was expressed as no education, primary, secondary, and higher.

The following factors were considered at community level: community level poverty, community level women's education, community distance to health facility, place of residence (urban or rural) and region. Region was used as provided in the NDHS dataset as Northcentral, Northeast, Northwest Southeast, South-south and Southwest. Other community level variables were computed by aggregating individual characteristics at the cluster level, dividing the measure into tertiles, and categorizing as low, medium and high. Similar procedure has been widely applied to derive community variables in DHS datasets [13,15]. Community level poverty was defined as the proportion of women who are from the poorest communities. Community women's education was defined as proportion of women from community with at least secondary education. Community distance to health facility was defined as the proportion of women for whom distance to health facility is a big problem, aggregated at cluster level.

Data analysis

Data analysis was done using Stata (17, StataCorp LLC, College Station, TX, USA). In DHS analysis, in order to adjust for multi-level cluster sampling design and non-response, individual women's survey weights are needed. Therefore, we adjusted for sampling weights, clustering, and stratification. Descriptive analysis included the trends analysis and frequency distribution to present background characteristics. Trend analysis was presented in bar charts.

Bivariate analysis was done using Chi-Square test to test the association between the independent variables and place of delivery. Taking into consideration the hierarchical structure of the DHS, a multilevel logistic regression analysis was done to identify the determinants of health facility delivery. Observations with missing data were excluded from the multivariate analysis. The variable marital status displayed collinearity and was not included in the multivariate analysis. To create spatial maps for health facility delivery coverage in Nigeria, a

sampling dataset was used and analysed using the QGIS 3.321 (https://qgis.org/en/site/). In order to normalize the dataset and make it easier to integrate into the database and visualize in QGIS, the data structure was created using Google Sheets. The attribute data was then combined with the spatial data using the Join Attribute by Location Tool in QGIS. This produced a database that contained the health facility delivery results along with the Nigeria shape file sourced from the Natural Earth database. To graphically represent each point of the attribute data for the variables in QGIS, the Equal Count (Quantile) mode and five classes were employed. The classes show the proportion of each attribute's data that is contained in the database, and this information was displayed using different colour ramps. This step is crucial in the data cleaning process and helps to ensure that the data is accurately represented in the visualization.

Ethical approval

Being a secondary data analysis, ethical approval was not required for this study. We registered and obtained permission to download the datasets from the measure DHS website. However, in the primary studies – The NDHS 2008, 2013, and 2018, the survey protocols were approved after review by the ICF Institutional Review Board and the National Health Research Ethics Committee of Nigeria (NHREC). Informed consent was obtained, and all methods were performed in accordance with the Declaration of Helsinki.

Results

Forty-one percent of women delivered their last child in a health facility. Most (49.26%) of the respondents were of the 20 – 29 years age group. Sixty-one percent were Muslims, 46.42% were of the Hausa/Fulani tribe, 44.44% had no formal education and 68.39% were employed. Majority (60.12%) of the respondents were not exposed to mass media, or had infrequent exposure, majority (87.85%) desired the index pregnancy, and more than half (57.79%) had at least four antenatal visits. Majority (83.60%) found companionship to the health facility as "not a big problem" but only 38.40% of the women participated in decisions regarding their health. Most of the women (60.24%) resided in rural areas. (Table 1)

Table 1: Sample Characteristics and Prevalence of Health Facility Delivery among Women of Reproductive Age in Nigeria. (NDHS 2018) (N = 21,792)

Variables	Frequency	Percentage
**Facility based delivery**		
Utilized health facility delivery	9001	41.14
Did not utilize health facility delivery	12791	58.86
**Age at last childbirth**		
15 – 19	2631	12.20
20 – 29	10685	49.26
30 – 39	7180	32.76
40 - 49	1296	5.781
**Marital status**		
Not married	1373	5.81
Married	20419	94.19
**Religion**		
Christianity	8929	38.08
Islam	12687	61.39
Traditional religion/others	176	0.53
**Ethnicity***		
Hausa/Fulani	9226	46.42
Yoruba	2357	12.61
Ibo	2836	12.62
Others	7355	28.35
**Level of education**		
No formal education	9527	44.44
Primary education	3410	15.03
Secondary education	7064	31.77
Higher	1791	8.76
**Employment status**		
Unemployed	6977	31.61
Employed	14815	68.39
**Wealth index**		
Poor	7264	31.72
Middle	7264	32.29
Rich	7264	35.99
**Exposure to mass media**		
No exposure/infrequent exposure	13,446	60.12
Frequent exposure	8346	39.88
**Wanted index pregnancy**		
Wanted	19054	87.85

Not wanted	2738	12.15
**Number of childbirths**		
1 - 2	7493	35.06
3 - 4	6233	28.16
≥ 5	8066	36.79
**Number of antenatal visits***		
< 4 visits	9158	42.21
At least 4 visits	12307	57.79
**Companionship to health facility**		
A big problem	3574	16.40
Not a big problem	18218	83.60
**Participates in healthcare decision**.		
Participates	8309	38.40
Does not participate	13483	61.60
**Partner's highest level of education***		
No formal education	7141	36.14
Primary education	2897	13.95
Secondary education	7060	34.46
Higher	3039	15.44
**Community poverty**		
Low	10292	48.89
Medium	4299	20.71
High	7201	30.41
**Community women's education**		
Low	7285	34.29
Medium	7253	32.73
High	7254	32.99
**Community distance to health facility**		
Low	7281	35.28
Medium	7319	33.54
High	7192	31.19
**Place of residence**		
Urban	7710	39.76
Rural	14082	60.24
**Region**		
Northcentral	3875	13.83
Northeast	4506	17.63
Northwest	6309	34.89
Southeast	2365	9.75
South-south	2174	9.21

Southwest	2563	14.69

*Variables with missing data

The proportion of women who delivered at a health facility increased from 2008 to 2018 for all regions, with exception of the South-south region where facility-based delivery remained almost constant in 2013 and 2018. The Northwest had the lowest prevalence of health facility delivery of 9.34% in 2008, 12.8% in 2013 and 16.36% in 2018, while the Southeast had the highest prevalence of 73.84% in 2008, 78.83% in 2013 and 80.8% in 2018. In Nigeria, health facility delivery increased slightly from 36.57% in 2008 to 37.44% in 2013 and then to 41.14% in 2018 (Figure 1).

Figure 1: Regional trends of health facility delivery in Nigeria (NDHS 2008, NDHS 2013, NDHS 2018)

Table 2 shows that all the independent variables showed statistically significant association with the outcome variable 'health facility delivery'. Higher proportion of women of the age group 20 – 29 years (41.37%) and 30 – 39 years (46.07%) delivered in a health facility compared to older women aged 40 – 49 years (36.03%) and younger women aged 15 – 19 years (29.44%). A higher proportion of Christian women (65.75%) than Muslim women (25.98%) and women of traditional religion (29.14%) delivered in a health facility. Igbo women had the highest utilization of health facility for delivery (81.30%), followed by Yoruba women (75.29%), then other minority tribes (46.81%) and Hausa/Fulani women (17.46%). While majority of women with higher than secondary education (87.71%) delivered in a health facility, only 15.14% of women without formal education delivered in a health facility. While 69.69% of women from the rich wealth tertile delivered in a health facility, only 13.99% of women from the poor wealth tertile delivered in a health facility (Table 2).

Table 2: Bivariate analysis of factors associated with health facility delivery among women of reproductive age in Nigeria (NDHS 2018) (N = 21,792)

**Variables**	**Facility based delivery**		**p-value**
	**Did not utilise health facility delivery**	**Utilised health facility delivery**	
	**Freq (%)**	**Freq (%)**	
**Age at last childbirth**			
15 – 19 years	1821 (70.56)	810 (29.44)	<0.0001
20 – 29 years	6289 (58.63)	4396 (41.37)	
30 – 39 years	3876 (53.93)	3304 (46.07)	
40 – 49 years	805 (63.97)	491 (36.03)	
**Marital Status**			
Not married	687 (48.09)	686 (51.91)	<0.0001
Married	12104 (59.52)	8315 (40.48)	
**Religion**			
Christianity	3306 (34.25)	5623 (65.75)	<0.0001
Islam	9357 (74.02)	3330 (25.98)	
Traditional	128 (70.86)	48 (29.14)	
**Ethnicity***			
Hausa/Fulani	7589 (82.54)	1637 (17.46)	<0.0001
Yoruba	550 (24.71)	1807 (75.29)	
Ibo	559 (18.7)	2277 (81.3)	
Others	4087 (53.87)	3268 (46.81)	
**Level of education**			
No formal education	8065 (84.86)	1462 (15.14)	<0.0001
Primary education	1960 (58.47)	1450 (41.53)	
Secondary education	2537 (35.49)	4527 (64.51)	
Higher	229 (12.29)	1562 (87.71)	
**Employment status**			
Unemployed	4878 (70.89)	2099 (29.11)	<0.0001
Employed	7913 (53.29)	6902 (46.71)	
**Wealth index**			
Poor	6171 (86.01)	1093 (13.99)	<0.0001
Middle	4446 (64.00)	2818 (36.00)	
Rich	2174 (30.31)	5090 (69.69)	
**Exposure to mass media**			
No exposure/infrequent exposure	9457 (71.42)	3989 (28.58)	<0.0001
Frequent exposure	3334 (39.90)	5012 (60.10)	
**Wanted index pregnancy**			
Wanted	11459 (60.38)	7595 (39.62)	<0.0001
Not wanted	1332 (47.79)	1406 (52.21)	

**Number of childbirths**			
1 – 2	3777 (49.59)	3716 (50.41)	<0.0001
3 – 4	3448 (55.34)	2785 (44.66)	
≥ 5	5566 (70.38)	2500 (29.62)	
**Number of antenatal visits***			
< 4 visits	7742 (85.03)	1416 (14.97)	<0.0001
At least 4 visits	4941 (40.69)	7366 (59.31)	
**Companionship to health facility**			
A big problem	2600 (70.31)	974 (29.69)	<0.0001
Not a big problem	10191 (56.72)	8027 (43.28)	
**Participates in healthcare decision**.			
Participates	3585 (42.13)	4724 (57.87)	<0.0001
Does not participate	9206 (69.28)	4277 (30.72)	
**Partner's highest level of education***			
No formal education	6236 (87.43)	905 (12.57)	<0.0001
Primary education	1719 (60.69)	1178 (39.31)	
Secondary education	3063 (43.03)	3997 (56.97)	
Higher	888 (28.87)	2151 (71.13)	
**Community poverty**			
Low	3733 (37.34)	6559 (62.66)	<0.0001
Medium	2974 (70.92)	1325 (29.08)	
High	6084 (85.23)	1117 (14.77)	
**Community education**			
Low	6544 (90.08)	741 (9.917)	<0.0001
Medium	4195 (58.38)	3058 (41.62)	
High	2052 (26.87)	5202 (73.13)	
**Community distance to health facility**			
Low	3453 (49.5)	3828 (50.5)	<0.0001
Medium	4132 (57.08)	3187 (42.92)	
High	5206 (71.35)	1986 (28.65)	
**Place of residence**			
Urban	2955 (37.88)	4755(62.12)	<0.0001
Rural	9838 (72.70)	4246 (27.30)	
**Region**			
Northcentral	1878 (49.63)	1997 (50.37)	<0.0001
Northeast	3337 (73.32)	1169 (26.68)	
Northwest	5320 (83.64)	989 (16.36)	
Southeast	478 (19.2)	1887 (80.80)	
South-south	1176 (49.1)	998 (50.90)	
South west	602 (23.8)	1961 (76.20)	

*Variables with missing data

A greater proportion of women with frequent exposure to mass media (60.10%) delivered in a health facility compared to those with infrequent or no exposure to mass media (28.58%). The proportion that reported delivery of their last child in a health facility was higher among women who did not desire the index pregnancy (52.21%) than for women who desired the index pregnancy (39.62%). The proportion that reported delivery of their last child in a health facility was higher among women with at least four antenatal visits (59.31%) than for women with less than four visits (14.97%). A greater proportion of women who reported companionship to health facility as not a big problem and those who participate in decisions regarding their health delivered in a health facility than women who reported companionship to health facility as a big problem and those who did not participate in decisions regarding their health, respectively.

The proportion of women who had their most recent birth in a health facility was highest for women whose partner had higher than secondary school education (71.13%) and lowest for women whose partners had no formal education. Communities with a low proportion of poor people, a low proportion of uneducated people, and a low proportion of people who considered distance to health facility as a big problem had higher rates of health facility delivery. A higher proportion of women in urban (62.12%) compared with women living in rural areas (27.30%) delivered in a health facility. The Northwest region had the lowest proportion (16.36%) of women delivering in health facilities while the Southeast region (80.80%) had the highest proportion of women delivering in health facilities. (Table 2)

In Model 4, the final model which consisted of individual and community variables, Ibo women had 3 times higher odds of delivering in a health facility than Hausa/Fulani women (AOR 3.08; 95% CI 2.11 – 4.49) and women from ethnic minorities had 50% higher odds of delivering in a health facility compared with Hausa/Fulani women (AOR 1.50; 95% CI 1.24 – 1.83). As level of education increased, the odds of delivering in a health facility also increased and similarly as partners level of education increased, the odds of delivering in a health facility increased. Compared with women from the poor wealth index, women of the middle wealth index had 32% higher odds of delivering in a health facility (AOR 1.32; 95% CI 1.12 – 1.54) and women of the rich wealth index had 90% higher odds of delivering in a health facility (AOR 1.90; 95% CI 1.55 – 2.33). Women with frequent exposure to mass media had 34% higher odds of delivering in a health facility (AOR 1.34; 95% CI 1.20 – 1.50). The odds of delivering in a health facility reduced as number of childbirths increased. Women with at least four antenatal visits were 4 times more likely to deliver in a health facility than women with less than four antenatal visits (AOR 4.03; 95% CI 3.51 – 4.62). Women who reported getting a companion to go the health facility was a big problem were less likely to deliver in a health facility (AOR 0.84; 95% CI 0.72 – 0.98). (Table 3)

Table 3: Multilevel analysis showing determinants of health facility delivery among women of reproductive age in Nigeria (NDHS 2018)

Variables	Model 1	Model 2	Model 3	Model 4
	Empty Model	Individual variables	Community variables	Individual/Community variables
		Adjusted Odds ratio	Adjusted Odds ratio	Adjusted Odds ratio
**Age at last childbirth**				
15 – 19 years		1		1
20 – 29 years		0.90 (0.76 – 1.07)		0.86 (0.72 – 1.02)
30 – 39 years		1.14 (0.93 – 1.41)		1.07 (0.87 – 1.32)
40 – 49 years		1.25 (0.95 – 1.65)		1.16 (0.88 – 1.53)
**Religion**				
Christianity		1		1
Islam		0.96 (0.81 – 1.14)		1.06 (0.89 – 1.27)
Traditional/others		0.62 (0.34 – 1.12)		0.87 (0.48 – 1.57)
**Ethnicity**				
Hausa/Fulani		1		1
Yoruba		4.15 (3.18 – 5.40) ***		1.21 (0.89 – 1.65)
Ibo		9.46 (6.90 – 12.98) ***		3.08 (2.11 – 4.49) ***
Others		2.25 (1.87 – 2.71) ***		1.50 (1.24 – 1.83) ***
**Highest level of education**				
No formal education		1		1
Primary education		1.36 (1.17 – 1.58) ***		1.24 (1.07 – 1.44) **
Secondary education		1.88 (1.60 – 2.20) ***		1.67 (1.42 – 1.95) ***
Higher		4.40 (3.36 – 5.76) ***		3.82 (2.92 – 4.99) ***
**Employment status**				
Unemployed		1		1
Employed		1.05 (0.93 – 1.17)		1.05 (0.94 – 1.18)
**Wealth index**				
Poor		1		1

Middle		1.71 (1.47 – 1.99) ***		1.32 (1.12 – 1.54) **
Rich		2.90 (2.39 – 3.50) ***		1.90 (1.55 – 2.33) ***
**Exposure to mass media**				
Not exposed/infrequent exposure		1		1
Frequent exposure		1.35 (1.21 – 1.51) ***		1.34 (1.20 – 1.50) ***
**Wanted index pregnancy**				
Wanted		1.17 (1.01 – 1.51) *		1.15 (0.99 – 1.34)
Not wanted		1		1
**Number of childbirths**				
1 – 2		1		1
3 – 4		0.77 (0.67 – 0.87) ***		0.77 (0.68 – 0.87) ***
≥ 5		0.67 (0.57 – 0.78) ***		0.68 (0.58 – 0.79) ***
**Number of antenatal visits**				
< 4 visits		1		1
At least 4 visits		4.17 (3.63 – 4.79) ***		4.03 (3.51 – 4.62) ***
**Companionship to health facility**				
A big problem		0.80 (0.69 – 0.93) **		0.84 (0.72 – 0.98) *
Not a big problem		1		1
**Participates in healthcare decision**.				
Participates		1.12 (1.00 – 1.25) *		1.09 (0.98 – 1.22)
Does not participate		1		1
**Partner's highest level of education**				
No formal education		1		1
Primary education		1.32 (1.11 – 1.56)		1.21 (1.02 – 1.44) *
Secondary education		1.56 (1.33 – 1.82)		1.43 (1.23 – 1.68) ***

Higher		2.35 (1.92 – 2.88)		2.14 (1.75 – 2.62) ***
**Community poverty**				
Low			1	1
Medium			0.52 (0.40 – 0.67) ***	0.74 (0.59 – 0.93) *
High			0.33 (0.24 – 0.44) ***	0.63 (0.48 – 0.83) **
**Community education**				
Low			1	1
Medium			3.73 (2.78 – 5.01) ***	1.72 (1.32 – 2.24) ***
High			12.33 (8.38 – 18.14) ***	2.65 (1.88 – 3.72) ***
**Community distance to health facility**				
Low			1	1
Medium			0.80 (0.69 – 0.97)	0.88 (0.73 – 1.05)
High			0.52 (0.41 – 0.67)	0.71 (0.56 – 0.88) **
**Place of residence**				
Urban			1	1
Rural			0.80 (0.65 – 0.97) *	1.03 (0.86 – 1.23)
**Region**				
Northcentral			1	1
Northeast			0.44 (0.33 – 0.58) ***	0.54 (0.42 – 0.71) ***
Northwest			0.18 (0.13 – 0.24) ***	0.25 (0.19 – 0.34) ***
Southeast			2.81 (2.01 – 3.91) ***	1.17 (0.77 – 1.78)
South-south			0.20 (0.15 – 0.28) ***	0.20 (0.14 – 0.27) ***
Southwest			1.52 (1.13 – 2.04) **	1.18 (0.86 – 1.62)
**Variance**	1.212 (0.751 – 1.957) ***	0.788 (0.424 – 1.465) ***	1.191 (0.742 – 1.810) ***	0.757 (0.401 – 1.431) ***
**ICC (%)**	71.69	39.11	44.93	35.20
**Log Likelihood**	-9723.7591	-8352.1517	-8998.7979	-8174.3727
**Model fit Statistics**				

**AIC**	19453.52	16758.30	18027.60	16426.75
**BIC**	19477.21	16971.46	18146.03	16734.64

Women from communities with medium level of community poverty (AOR 0.74; 95% CI 0.59 – 0.93). and those from communities with high level off community poverty (AOR 0.63; 95% CI 0.48 – 0.83) had lesser odds of delivering in a health facility, compared to women from communities with low level of community poverty. Compared with women from communities with low level of female education, women from communities with medium level of female education (AOR 1.72; 95% CI 1.32 – 2.24). and those from communities with high level of female education (AOR 2.65; 95% CI 1.88 – 3.72) had higher odds of delivering in a health facility. Women from communities where a high proportion of women considered distance to health facility as a big problem were less likely to use health facility for child delivery (AOR 0.71; 95% CI 0.56 – 0.88). Women from the Northeast, Northwest and South-south regions were less likely to use health facility for child delivery, compared with women from the Northcentral region. (Table 3)

Age at last childbirth, religion, employment status, wanted index pregnancy, participation in healthcare decision and place of residence did not show statistically significant relationship with place of delivery. (Table 3)

Figure 2: Percentile map showing the prevalence of health facility delivery among women of reproductive age in Nigeria (NDHS 2018)

Discussion

This study assessed the regional trends, determinants, and spatial patterns of health facility delivery in Nigeria. Our study reveals that only four in ten of women deliver in health facility in the country. This finding is significant as it demonstrates that poor facility delivery among women of reproductive age exists in the country and is in keeping with the findings of Bolarinwa et al., who similarly used the NDHS to assess health facility delivery among women of reproductive age [7]. In addition, this finding is equally essential because the place of delivery impacts the quality of maternal and child health services received and may thus emphasize the need for improving existing strategies and establishment of new programmes to improve the uptake of facility delivery among reproductive-age women.

Our study is the first study in Nigeria to assess the trends of health facility delivery across the six geopolitical zones. Fasina et. al examined the trends of health facility delivery in Nigeria across two time points – NDHS 2013 and NDHS 2018 for women of reproductive age and found an increase from 38.02% to 42.04% [17]. Our study showed a similar finding of 36.57% in 2008, 37.44% in 2013 and 41.14% in 2018. The slight differences in result could be adduced in differences in the management of variables e.g., handling of variables with missing values. A study from Kenya showed higher prevalence of health facility delivery compared to our study, reporting an increase from 68.3% in 2003 to about 95% in 2015 [18]. In Senegal, facility-based delivery rose from 47% in 1993 to 73% in 2014 and in Namibia, it rose from 67% in 1992 to 87% in 2013 [19]. Despite the positive trend observed in our study, the prevalence of health facility delivery is much lower than these African countries. Even though the efforts to improve maternal healthcare utilization is yielding results in Nigeria, there need for more intensified efforts to improve health facility delivery and ultimately delivery outcomes.

From our study, all geopolitical zones showed an increase in health facility delivery prevalence across the years, with exemption of South-south zone where the prevalence of health facility delivery remained relatively the same in 2013 and 2018. Prevalence of health facility delivery was least in the northern geopolitical zones and the South-south. These may be due to higher levels of poverty, illiteracy, and sociocultural beliefs that may contribute to the low utilization of health facilities for delivery.

On conducting bivariate analysis, we found that all explanatory variables were significantly associated with health facility delivery. On further analysis, the study found that as the level of education increased, the women were more likely to choose health facility delivery. This finding is consistent with previous studies, including those that analysed the DHS across the African sub-continent [20-24]. Education has been shown to promote women's independence in making better health decisions, and highly educated mothers are more likely to seek better health services. Low maternal education has been a significant barrier to health facility delivery and seeking skilled attendants during childbirth [20,24]. Programmes to improve health literacy among women can improve the use of health facilities for delivery while promoting and ensuring the education of the girl child and women. Another finding of this study is the significant association between wealth index and health facility delivery. As the household wealth index of women increased, the likelihood of facility delivery also increased, this result is in agreement with earlier studies from Nigeria and Ghana [7,22]. One possible explanation for this finding is that poorer households are prone to financial constraints that may hinder care seeking at health facilities with skilled attendants at delivery. To reduce disparities in access to healthcare, it is critical to reduce financial barriers for pregnant women. This can be achieved through a functional and efficient health insurance scheme and support for poor women to access health facility during pregnancy.

Access to media also had a significant association with health facility delivery, with the probability of facility delivery increasing with access to media. Pregnant women who have access to media are more likely to obtain valuable information on the benefits of health facility delivery. This finding suggests that the media plays a significant role in providing education and health information, which can considerably influence health-seeking behaviour.

It was found that the probability of health facility delivery reduced as number of childbirths increased. The finding agrees with that of a study from East Africa [25]. The significance of these findings is that women with more children may assume themselves to be more experienced with childbirth, making them choose to deliver at home rather than seek skilled delivery. Another possible reason could be that having a large family size means fewer resources for seeking healthcare, not only for the children within the household but also for the pregnant mother herself. Additionally, if women had a negative experience with health workers during a previous health facility delivery, they may choose to avoid having another health facility delivery. Therefore, it is crucial to provide proper training and supervision for health workers to ensure they demonstrate the right conduct in their provision of maternal care.

Our study revealed that women who had at least four ANC visits were more likely to deliver in the health facility, in keeping with findings from other studies [26,27]. Optimal ANC attendance provides the opportunity for the women to receive information on available maternal health care services and the benefits of utilizing them, unlike women with no such opportunity. Similarly, our study found that women who reported getting support to go to a health facility was a big problem had a lower probability of delivering in a health facility. Spousal and family support can improve health-seeking for facility delivery and utilization of maternal services health facilities.

Our study also established that women from communities with medium and high community poverty had reduced probabilities of delivering in a health facility, unlike those from communities with low community poverty. Likewise, compared with women from communities with low levels of female education, women from communities with medium levels of female education and those from communities with high levels of female education had higher possibilities of delivering in a health facility. This finding is in consistent with that of a previous study [20]. This finding reveals the need for programmes and interventions that can reduce community poverty and improve communities' socioeconomic status and education level. The study found that women from communities who reported distance to health facilities as a big problem had lower prospects of health facility delivery in keeping with findings of previous studies [7,20]. A possible explanation for this finding is that when health facilities are not easily accessible, ready alternatives are likely to be preferred to avoid the anticipated difficulties associated with seeking maternal health services from health facilities.

Further still, it was observed in this study that women from the Northeast, Northwest and South-south regions were less likely to utilize health facilities for delivery when compared to women from the Northcentral region of the country. This can also be observed from the spatial analysis in which unlike the north, the southwestern and southeastern states had high and very high utilization of health facility for delivery, and this was seen to decrease moving towards the northern part of the country. These findings agree with that of a previous study [12] and could possibly be due to contextual disparities. These regions have varying levels of poverty, illiteracy, and sociocultural beliefs that may contribute to the findings [28].

In addressing the findings from this study, the government, at all levels, must focus on individual and community-level issues that contribute to the low uptake of health facility

delivery among women through a combination of approaches with particular attention to context-specific solutions that address the factors contributing to these findings.

Strengths and Limitations

This study is the first to examine the trends of health facility delivery across geopolitical zones in Nigeria and is also the first study to conduct a spatial analysis of health facility delivery in the country. However, there are still some limitations to be acknowledged. Firstly, the data was self-reported and collected retrospectively, thus study is susceptible to recall bias. Secondly, the study dataset was cross-sectional in nature, it was only possible to establish an association and not causality. Thirdly, due to multicollinearity, we couldn't determine the effect of marital status on health facility delivery.

Conclusion

The current study revealed the low prevalence of health facility delivery among women of reproductive age in Nigeria. It further showed the influence of factors such as achieving higher levels of education, having fewer children, having optimal ANC attendance, and many other factors have on health facility delivery. There is a need to deploy appropriate strategies and programme to improve health facility delivery.

References

1. Gebreegziabher SB, Marrye SS, Kumssa TH, Merga KH, Feleke AK, Dare DJ et al. Assessment of maternal and child health care services performance in the context of COVID-19 pandemic in Addis Ababa, Ethiopia: evidence from routine service data. Reprod Health. 2022 Feb 14;19(1):42. doi: 10.1186/s12978-022-01353-6.
2. Ossai EN, Eze II, Eke PC, Onah CK, Agu C, Ogbonnaya LU. Where, why and who delivers our babies? Examining the perspectives of women on utilization of antenatal and delivery services in a developing country. BMC Pregnancy Childbirth. 2023 Jan 2;23(1):1. doi: 10.1186/s12884-022-05306-6.
3. Zhang X, Anser MK, Ahuru RR, Zhang Z, Peng MY, Osabohien R, Mirza M. Do Predictors of Health Facility Delivery Among Reproductive-Age Women Differ by Health Insurance Enrollment? A Multi-Level Analysis of Nigeria's Data. Front Public Health. 2022 Apr 14;10:797272. doi: 10.3389/fpubh.2022.797272.
4. Eluobaju D, Okonofua F, Weine S, Goba G. Understanding birthing preferences of women in Benin City, Nigeria: a qualitative study. BMJ Open. 2023 May 2;13(5):e054603. doi: 10.1136/bmjopen-2021-054603
5. UNICEF. Delivery care. Available from: https://data.unicef.org/topic/maternal-health/delivery-care/ (Accessed 13/08/2023)
6. Michael T. O, Nwokocha E. E. and Ukwandu D. Child Delivery Care Practices among Unmarried Younger Adolescents in Nigeria: The Case of Akwa Ibom State. NJESS. 2021; 63(3):403-432.
7. Bolarinwa OA, Fortune E, Aboagye RG, Seidu AA, Olagunju OS, Nwagbara UI, Ameyaw EK, Ahinkorah BO. Health facility delivery among women of reproductive age in Nigeria: Does age at first birth matter? PLoS One. 2021 Nov 4;16(11):e0259250. doi: 10.1371/journal.pone.0259250.

8. National Population Commission (NPC) [Nigeria] and ICF. 2019. 2018 Nigeria DHS Key Findings. Abuja, Nigeria and Rockville, Maryland, USA: NPC and ICF.
9. UN Department of Economic and Social Affairs Sustainable Development, United Nations. The 17 Goals Available from:https://sdgs.un.org/goals (Accessed 13/08/2023)
10. Maternal Health Task Force. Five ways an innovative program increased facility birth in Nigeria. Available from: https://www.mhtf.org/2015/01/13/five-ways-an-innovative-program-increased-facility-birth-in-nigeria/ (Accessed 13/08/2023)
11. UCSF. Strategies to increase health facility deliveries: Three case studies. 2014; Available from: https://globalhealthsciences.ucsf.edu/global-health-group/%0Aghg-publications (Accessed 13/08/2023)
12. Ononokpono DN, Odimegwu CO. Determinants of Maternal Health Care Utilization in Nigeria: a multilevel approach. Pan Afr Med J. 2014 Jan 18;17 Suppl 1(Suppl 1):2. doi: 10.11694/pamj.supp.2014.17.1.3596.
13. Adedokun ST, Uthman OA. Women who have not utilized health Service for Delivery in Nigeria: Who are they and where do they live? BMC Pregnancy Childbirth. 2019;19(1):1–14.
14. Dahiru T, Oche OM. Determinants of antenatal care, institutional delivery and postnatal care services utilization in Nigeria. Pan Afr Med J. 2015;21:1–17.
15. Lukman Solanke B, Adebayo Rahman S, Olasupo Ogunjuyigbe P. To what extent do community characteristics drive health facility delivery? Findings among Women who had recent live births in Nigeria. Heal Soc Care Community. 2021;29(4):992–1000.
16. USAID. The DHS Program - Demographic and Health Survey (DHS). Available from: https://dhsprogram.com/Methodology/Survey-Types/DHS.cfm (Accessed 20/08/2023)

17. Fasina F, Oni G, Oduaran A, Samuel G. Trends in maternal health facilities utilization and women's autonomy in Nigeria (2013-2018): Attainment of sustainable development goals-5, Cogent Arts & Humanities. 2020; 7:1, doi: 10.1080/23311983.2020.1823598

18. Atahigwa C, Kadengye DT, Iddi S, Abrams S, Rie AV. Trends and determinants of health facility childbirth service utilization among mothers in urban slums of Nairobi, Kenya. Global Epidemiology.2020;2:100029

19. Udo IE, Doctor HV. Trends in Health Facility Births in sub-Saharan Africa: An Analysis of Lessons Learned under the Millennium Development Goal Framework. Afr J Reprod Health. 2016 Sep;20(3):108-117.

20. Olubodun T, Rahman SA, Odukoya OO, Okafor IP, Balogun MR. Determinants of health facility delivery among young mothers aged 15 - 24 years in Nigeria: a multilevel analysis of the 2018 Nigeria demographic and health survey. BMC Pregnancy Childbirth. 2023 Mar 17;23(1):185. doi: 10.1186/s12884-023-05492-x.

21. Olusanya BO, Alakija OP, Inem VA. Non-uptake of facility-based maternity services in an inner-city community in Lagos, Nigeria: an observational study. J Biosoc Sci. 2010; 42:341-358 doi:10.1017/ S0021932009990526

22. Dankwah E, Zeng W, Feng C, Kirychuk S, Farag M. The social determinants of health facility delivery in Ghana. BMC Reprod Heal. 2019;16:1–10.

23. Zegeye B, Ahinkorah BO, Idriss-wheelr D, Oladimeji O, Olorunsaiye CZ, Yaya S. Predictors of institutional delivery service utilization among women of reproductive age in Senegal : a population-based study. Public Health. 2021;79:5.

24. Dimbuene ZT, Amo-Adjei J, Amugsi D, Mumah J, Izugbara CO, Beguy D. Women's education and utilization of maternal health services in Africa: A multi-country and socioeconomic status analysis. J Biosoc Sci. 2018;50(6):800–22.

25. Agaba P, Magadi M, Orton B. Predictors of health facility childbirth among unmarried

and married youth in Uganda. PLoS ONE. 2022;17(4):e0266657.

26. Nahar MT, Anik SMF, Islam MA, Islam SMS. Individual and communitylevel factors associated with skilled birth attendants during delivery in Bangladesh: A multilevel analysis of demographic and health surveys. PLoS ONE. 2022;17(6):e0267660.

27. Solanke BL, Rahman SA. Multilevel analysis of factors associated with assistance during delivery in rural Nigeria: Implications for reducing rural-urban inequity in skilled care at delivery. BMC Pregnancy Childbirth. 2018;18(1):1–15.

28. Jaiyeola AO, Choga I. Assessment of poverty incidence in Northern Nigeria. J Poverty. 2021;25(2):155–72.

Figure 1

Click here to access/download;Figure;Figure 1.tiff

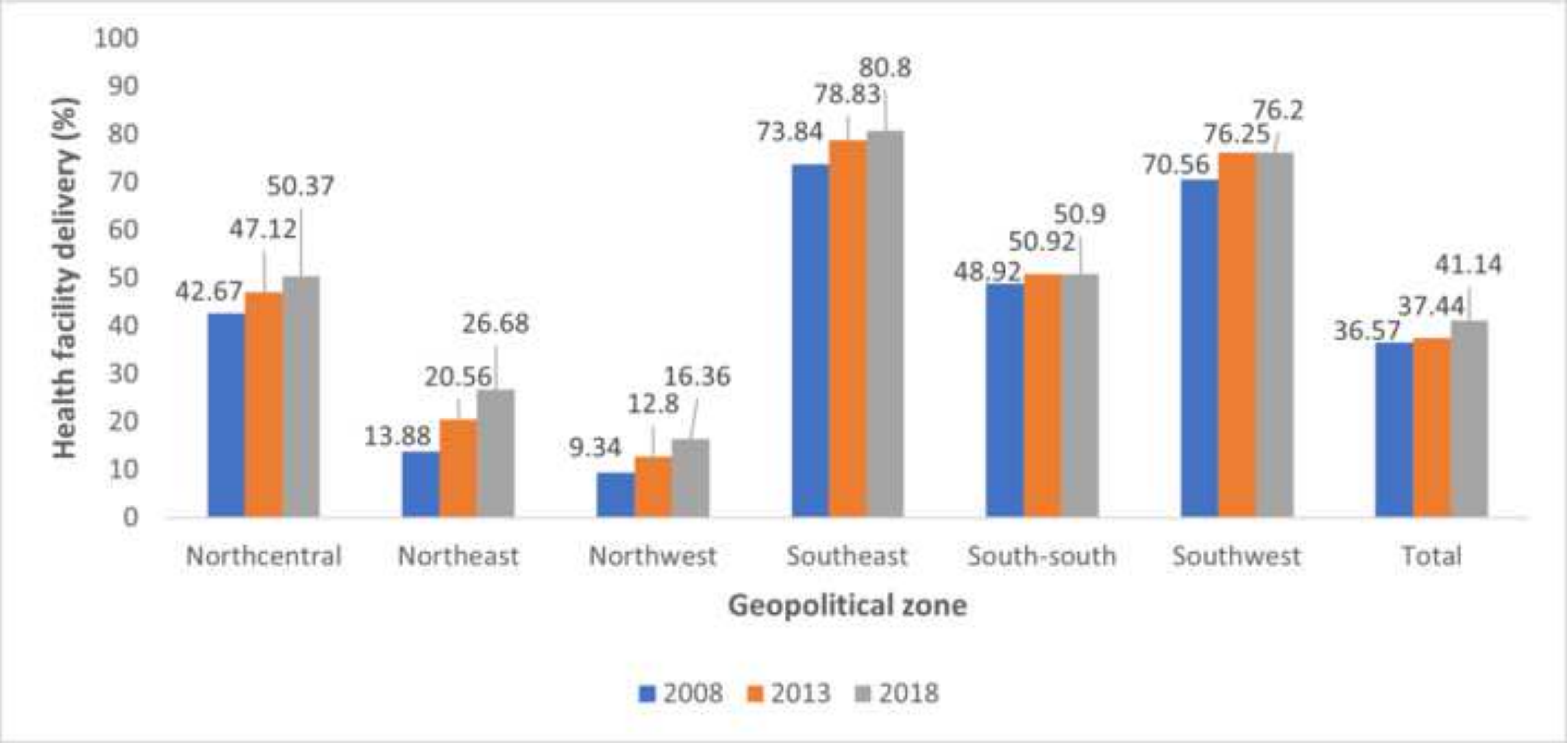

Figure 2 Click here to access/download;Figure;Figure 2.tiff

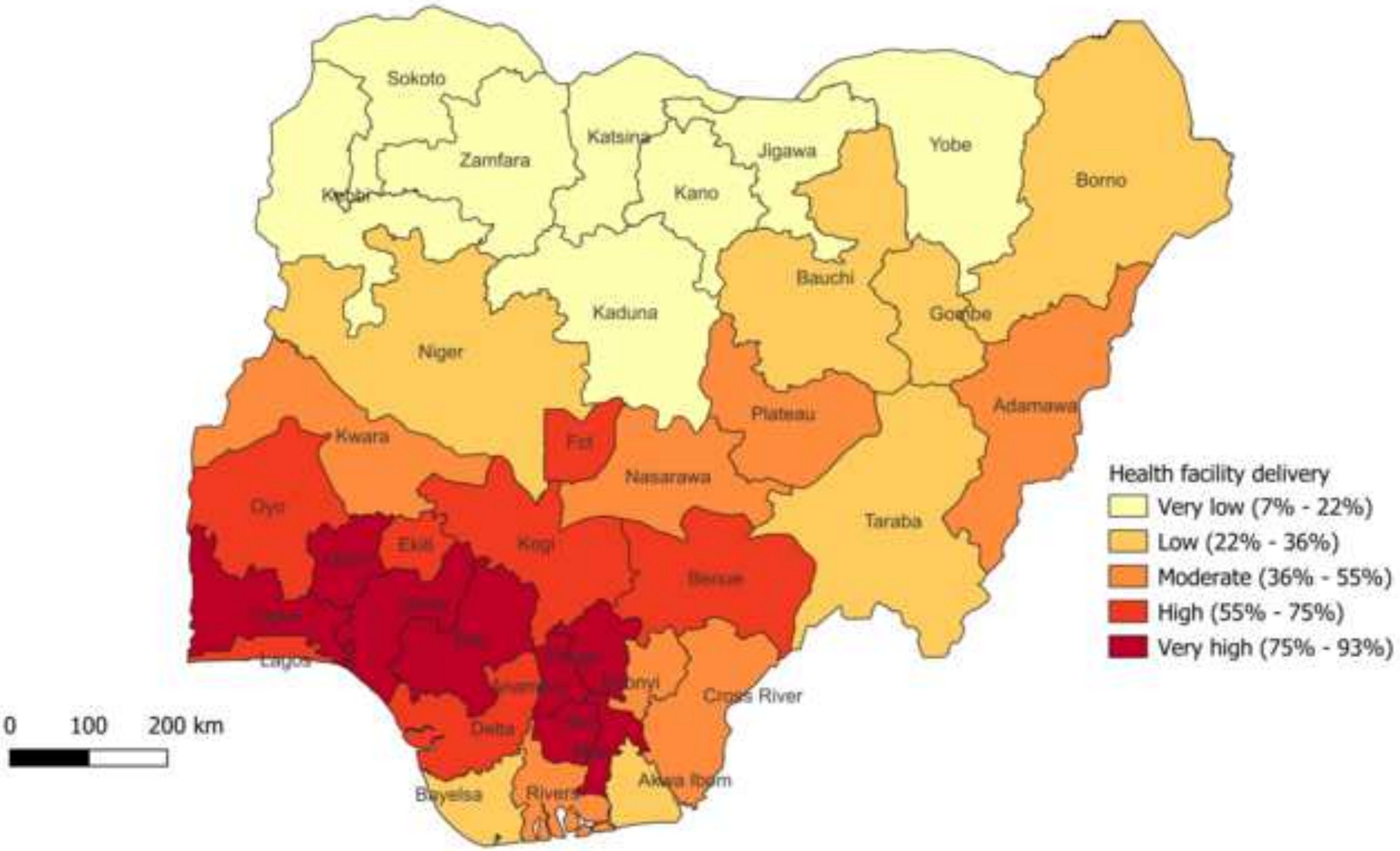

Supplement: Attachment — Submitted filename: PONE-D-23-37668 jzc_comments.pdf [file pone.0312005.s002.pdf]
