# Supplementary material for: Regional trends, spatial patterns and determinants of health facility delivery among women of reproductive age in Nigeria: A national population based cross-sectional study
Source: PLoS One. 2024 Oct 16;19(10):e0312005. doi: 10.1371/journal.pone.0312005 (PMC11482673; doi:10.1371/journal.pone.0312005)
Supplement: Attachment — Submitted filename: Response to comments.docx [file pone.0312005.s003.docx]

**COMMENT 1**

We note that Figure 2 in your submission contain map/satellite images which may be copyrighted. All PLOS content is published under the Creative Commons Attribution License (CC BY 4.0), which means that the manuscript, images, and Supporting Information files will be freely available online, and any third party is permitted to access, download, copy, distribute, and use these materials in any way, even commercially, with proper attribution. For these reasons, we cannot publish previously copyrighted maps or satellite images created using proprietary data, such as Google software (Google Maps, Street View, and Earth). For more information, see our copyright guidelines: <http://journals.plos.org/plosone/s/licenses-and-copyright>.

We require you to either (1) present written permission from the copyright holder to publish these figures specifically under the CC BY 4.0 license, or (2) remove the figures from your submission:

a. You may seek permission from the original copyright holder of Figure 2 to publish the content specifically under the CC BY 4.0 license.

We recommend that you contact the original copyright holder with the Content Permission Form (<http://journals.plos.org/plosone/s/file?id=7c09/content-permission-form.pdf>) and the following text:
“I request permission for the open-access journal PLOS ONE to publish XXX under the Creative Commons Attribution License (CCAL) CC BY 4.0 (<http://creativecommons.org/licenses/by/4.0/>). Please be aware that this license allows unrestricted use and distribution, even commercially, by third parties. Please reply and provide explicit written permission to publish XXX under a CC BY license and complete the attached form.”

Please upload the completed Content Permission Form or other proof of granted permissions as an ""Other"" file with your submission.

In the figure caption of the copyrighted figure, please include the following text: “Reprinted from [ref] under a CC BY license, with permission from [name of publisher], original copyright [original copyright year].”

b. If you are unable to obtain permission from the original copyright holder to publish these figures under the CC BY 4.0 license or if the copyright holder’s requirements are incompatible with the CC BY 4.0 license, please either i) remove the figure or ii) supply a replacement figure that complies with the CC BY 4.0 license. Please check copyright information on all replacement figures and update the figure caption with source information. If applicable, please specify in the figure caption text when a figure is similar but not identical to the original image and is therefore for illustrative purposes only.
The following resources for replacing copyrighted map figures may be helpful:

**RESPONSE 1**

The authors thank the journal office for this comment. The Nigeria shape file was sourced from the humanitarian data exchange (HDX)(<https://data.humdata.org/dataset/cod-ab-nga>?). We have contacted this source with the request for permission for the open-access journal PLOS ONE to publish the figure under the Creative Commons Attribution License (CCAL) CC BY 4.0, as stated above, and also provided the content permission form.

We however got a response that The HDX is an open data platform managed by the [United Nations Office for the Coordination of Humanitarian Affairs](https://www.unocha.org/) (OCHA) through its [Centre for Humanitarian Data](https://centre.humdata.org/) and dataset obtained therein are free for use. We have therefore included this in the manuscript under the subheading – Spatial Analysis (Line 238-241). We have also indicated the source of the shapefile just beneath the Figure 2 title (Line 371)

**COMMENT 2**

We are unable to open your Supporting Information file [STATA Do File.do]. Please kindly revise as necessary and re-upload."

**RESPONSE 2**

The STATA Do File has now been submitted in pdf format

NB: ABOVE ARE THE ONLY CHANGES MADE TO THE MANUSCRIPT. THERE ARE NO OTHER CHANGES TO THE MANUSCRIPT.
